# Prevalence of Ventricular Arrhythmia and Its Associated Factors in Nondialyzed Chronic Kidney Disease Patients

**DOI:** 10.1371/journal.pone.0066036

**Published:** 2013-06-07

**Authors:** Fabiana Oliveira Bastos Bonato, Marcelo Montebello Lemos, José Luiz Cassiolato, Maria Eugênia Fernandes Canziani

**Affiliations:** 1 Division of Nephrology, Department of Internal Medicine, Federal University of São Paulo, São Paulo, Brazil; 2 Cardios Research Institute, São Paulo, Brazil; S.G.Battista Hospital, Italy

## Abstract

**Background and Objectives:**

Sudden cardiac death is the most common cause of mortality in chronic kidney disease patients, and it occurs mostly due to ventricular arrhythmias. In this study, we aimed at investigating the prevalence of ventricular arrhythmia and the factors associated with its occurrence in nondialyzed chronic kidney disease patients.

**Design, Setting, Participants and Measurements:**

This cross-sectional study evaluated 111 chronic kidney disease patients (estimated glomerular filtration rate 34.7±16.1 mL/min/1.73 m^2^, 57±11.4 years, 60% male, 24% diabetics). Ventricular arrhythmia was assessed by 24-hour electrocardiogram. Left ventricular hypertrophy (echocardiogram), 24-hour ambulatory blood pressure monitoring, and coronary artery calcification (multi-slice computed tomography) and laboratory parameters were also evaluated.

**Results:**

Ventricular arrhythmia was found in 35% of the patients. Non-controlled hypertension was observed in 21%, absence of systolic decency in 29%, left ventricular hypertrophy in 27%, systolic dysfunction in 10%, and coronary artery calcification in 49%. Patients with ventricular arrhythmia were older (p<0.001), predominantly men (p = 0.009), had higher estimated glomerular filtration rate (p = 0.03) and hemoglobin (p = 0.005), and lower intact parathyroid hormone (p = 0.024) and triglycerides (p = 0.011) when compared to patients without ventricular arrhythmia. In addition, a higher left ventricular mass index (p = 0.002) and coronary calcium score (p = 0.002), and a lower ejection fraction (p = 0.001) were observed among patients with ventricular arrhythmia. In the multiple logistic regression analysis, aging, increased hemoglobin levels and reduced ejection fraction were independently related to the presence of ventricular arrhythmia.

**Conclusions:**

Ventricular arrhythmia is prevalent in nondialyzed chronic kidney disease patients. Age, hemoglobin levels and ejection fraction were the factors associated with ventricular arrhythmia in these patients.

## Introduction

Sudden cardiac death is the single most common cause of mortality in chronic kidney disease (CKD) patients undergoing dialysis, accounting for 20–30% of deaths [1]. A large database study has recently demonstrated that mortality attributed to sudden cardiac death was 14-fold increased among dialysis patients when compared to the general population, while the proportion of deaths from other cardiovascular complications was similar [2]. In CKD patients with documented coronary artery disease, the decrement of glomerular filtration rate (GFR) was shown to be a predictor of sudden cardiac death. Each 10 ml/min decrease in GFR was associated with 11% increase in the risk for sudden cardiac death. Additionally, while for patients with GFR ≥60 ml/min the sudden cardiac death rate was 3.8 per 1000 patient-years, the rate rose to 7.3 for patients with GFR 15–59 ml/min [3].

Epidemiological and observational studies have demonstrated that overall incidence of sudden cardiac death in CKD population is indeed greater than the incidence of coronary events [4], suggesting a worrisome increase in the frequency of ventricular arrhythmia, considered the foremost cause of sudden cardiac death. Few studies, however, have investigated the occurrence of ventricular arrhythmia in CKD populations. Data coming from our group have previously demonstrated that the frequency of ventricular arrhythmia was 48% in patients on hemodialysis [5], 45% in patients on peritoneal dialysis [6], and 30% among incident kidney transplant recipients [7].

The traditional view of ventricular arrhythmias pathophysiology posits a vulnerable diseased myocardium with a transient trigger. In individuals without CKD, the substrate for a terminal arrhythmia is most often an ischemic myocardium due to ruptured arterial plaque, a focal myocardial scar or a reduced left ventricular ejection fraction [8]. It is unknown whether this goes true for CKD patients, who have more frequently diastolic dysfunction, electrolyte disturbances and disorders in the mineral metabolism [9]. Studies are required to better characterize the associated risk factors for ventricular arrhythmia in CKD population.

Although cardiovascular mortality has shown to be substantially elevated since the early stages of CKD, the occurrence of ventricular arrhythmia and its associated risk factors has not been so far investigated in CKD patients not requiring dialysis. Thus, this study aimed at examining the prevalence of ventricular arrhythmia and investigating the factors associated with ventricular arrhythmia in nondialyzed CKD patients.

## Materials and Methods

### Population

A total of 111 non-dialyzed patients with CKD stages 2 to 5 were recruited from the outpatient clinic of the Federal University of São Paulo, São Paulo, Brazil. Patients on treatment for at least 3 months were approached to participate in the study. Exclusion criteria included age less than 18 years, presence of chronic inflammatory disease, active malignancy, human immunodeficiency virus, viral hepatitis, and chronic use of steroids. The majority of the patients were on regular use of angiotensin-converting enzyme inhibitors (81%) and diuretics (76%). Patients were also under use of β-blockers (44%), calcium channel blockers (40%), statins (32%) and angiotensin receptor blockers (22%). Thirty five patients (33%) were using sevelamer, six patients (5%) were taking calcium-based phosphate binders, and six patients (5%) were taking calcitriol. Five patients were using erythropoiesis-stimulating agents.

Written informed consent was obtained from all participants. This study was reviewed and approved by the Ethics Advisory Committee of the Federal University of Sao Paulo (approval number 60806).

### Study design and protocol

In this cross-sectional study all patients underwent clinical history assessment, laboratory tests and cardiac evaluation within a month. Demographic data, cardiovascular risk factors, comorbidities and family history were also evaluated. Nutritional status was evaluated by the subjective global assessment [10].

### Laboratory tests

Blood samples were drawn in a 12-hour fasting state. Biochemical and hematological parameters included serum creatinine, hemoglobin, potassium, magnesium, lipid profile, ionized calcium, phosphate, alkaline phosphatase, intact parathyroid hormone (iPTH - chemiluminescence imunoassay; Immulite; DPC-Biermann, Bad Nauheim, Germany ; reference values 10 to 65 pg/ml) and Fibroblast growth factor 23 (FGF23 - ELISA Kainos Laboratories, Tokyo, Japan). High-sensitivity C-reactive protein was determined by immunochemiluminescence (CRP Immunolite; Immunometric Assay, CA, USA) and interleukin-6 (IL-6) was measured using a commercially available enzyme-linked immunosorbent assay (BD Biosciences Pharmingen, CA, USA). Proteinuria was measured by obtaining 24-hour urine samples and abnormal proteinuria was defined as urinary protein excretion >150 mg/24 h. The glomerular filtration rate (eGFR) was estimated by the CKD-EPI (Chronic Kidney Disease Epidemiology Collaboration) equation [11]. The diagnosis and classification of CKD were established as described elsewhere [12].

### 24-hour electrocardiogram

Ventricular arrhythmia and supraventricular arrhythmia were evaluated by a 3-channel 24-hour electrocardiogram monitoring (Cardios-Light®, Cardios, São Paulo, Brazil). Ventricular arrhythmia was defined as the presence of ventricular extra-systoles.

### Echocardiogram

Two-dimensional color Doppler echocardiogram (Philips® HDI 5000, Royal Philips Electronics, Netherlands) was performed according to the recommendations of the American Society of Echocardiography [13]. Presence of left ventricular hypertrophy was considered for a left ventricular mass index ≥134 g/m^2^ among men and >110 g/m^2^ among women. Systolic dysfunction was defined as ejection fraction ≤55%.

### 24-hour ambulatory blood pressure monitoring

The 24-hour blood pressure monitoring was performed using *Dyna* equipment (Cardios, São Paulo, Brazil). Oscillometer was adjusted to systolic blood pressure varying between 290 and 70, and diastolic blood pressure varying between 180 and 45 mmHg, and memory of up to 300 measurements/events. Blood pressure (BP) measurements were obtained at intervals of 20 minutes during the day and 30 minutes during sleep. Participants were instructed to keep their habitual routine during the 24-h period and to pause momentarily during each BP measurement. Dipping (%) is defined by percent decrease in nighttime systolic-diastolic BP blood pressure compared to daytime systolic-diastolic BP. When patients exhibited dipping of less than 10%, they were defined as non-dippers [14]. Hypertension was defined as blood pressure greater than 140/90 mmHg or use of antihypertensive medication.

### Coronary computed tomography

Patients underwent coronary artery calcification (CAC) quantification by a multi-slice computed tomography scanner (LightSpeed® Pro 16; GE Healthcare, Milwaukee, WI, USA), using a gantry rotation of 0.4 seconds, collimation of 2.5 mm (slice thickness), and reconstruction time of six frames per second. A calcium threshold of 130 or more Hounsfield Units was used. The images were scored by a single radiologist blinded to the clinical and biochemical aspects of the patient. As described by Agatston et al. [15], the calcium score was determined by multiplying the area of each calcified lesion by a weighting factor corresponding to the peak pixel intensity for each lesion. The sum of each lesion of all coronary arteries was used for analysis. Presence of calcification was defined as CAC score >10 Agatston units (AU) and severe calcification as CAC score ≥400 AU.

### Statistical Analysis

Data were reported as mean and standard deviation (SD), median and interquartile range, or frequencies (proportions). Comparisons among continuous variables were done by Student's *t*-test and the Mann-Whitney U-test for normally distributed data and skewed data, respectively. The study population was further divided considering the presence of arrhythmia. Comparisons of proportions were done by chi-square analysis or by the Fisher exact test, when appropriate. The stepwise logistic regression analysis was applied to assess the factors associated with the presence of ventricular arrhythmia. All the variables with significance at p<0.05 level in the univariate analysis were considered in the multiple regression analysis. Statistical analysis was performed using SPSS for Windows (version 19; SPSS, Chicago, IL).

## Results

This study included 111 nondialyzed CKD patients, whose majority was middle-aged men. Demographic, laboratorial and cardiovascular data of the total population are summarized in Table 1. Patients had been on treatment for a median time of 2 years. Most of them were in stage *IIIa* (15%), stage *IIIb* (30%) or stage *IV* (41%) of CKD. The main CKD causes were hypertension and diabetes. Overweight and obesity were found in 32% and 27% of the patients, respectively. Malnutrition was observed in only 4% of the patients according to the subjective global assessment. Twenty-four percent of the patients had diabetes. Non controlled hypertension was observed in 21% of the patients, while absence of systolic decency in 29%. Left ventricular hypertrophy was found in 27% of the patients and systolic dysfunction in 10%. Coronary artery calcification was observed in 49%, from which 46% had severe calcification.

**Table 1:** General characteristics of the study population.

	(N = 111)
**Male [n(%)]**	67 (60%)
**Age (years)**	57±11.38
**Black [n(%)]**	21 (19%)
**Follow up time (months)**	21 (9–55)
**Diabetes [n(%)]**	27 (24%)
**Tobacco use [n(%)]**	57 (51%)
**Body mass index (kg/m^2^)**	26.8±5.26
**Creatinine (mg/dL)**	2.26±0.84
**eGFR (ml/min/1,73 m^2^)**	34.7±16.1
**Proteinuria (g/24 h)**	0.24 (0–0.79)
**Hemoglobin (g/dL)**	12.7±1.8
**Potassium (mEq/L)**	4.7 (4.3–5.1)
**Magnesium (mEq/L)**	1.9 (1.7–2.1)
**Ionized calcium (mmol/L)**	1.28±0.05
**Phosphate (mg/dL)**	3.78±0.72
**Alkaline phosphatase (mg/dl)**	81 (66–103)
**PTH (pg/ml)**	110 (63–193)
**iFGF 23 (pg/ml)**	47.3 (23.2–102.8)
**CRP (mg/dl)**	0.28 (0.12–0.77)
**IL6 (pg/ml)**	4.6 (2.7–8.4)
**Total cholesterol (mg/dL)**	184.2±37.7
**LDL cholesterol (mg/dL)**	101±28.2
**HDL cholesterol (mg/dL)**	51.5±14.3
**Triglycerides (mg/dL)**	125 (99–206)
**Median systolic pressure (mmHg)**	125 (116.7–137)
**Mean diastolic pressure (mmHg)**	78.6±10.9
**Absence of systolic decency [n(%)]**	32 (29%)
**Non controlled hypertension [n(%)]**	23 (21%)
**Left ventricular mass index (g/m^2^)**	102.3 (84.4–131.3)
**Ejection fraction (%)**	67 (62–70)
**Calcium score (AU)**	9 (0–334)

eGFR - estimated Glomerular Filtration Rate; iPTH - intact Parathyroid Hormone; FGF23 - Fibroblast Growth Factor 23; CRP - C-Reactive Protein; IL6 - Interleukin-6. Results in mean ± SD, median (interquartiles) or proportions.

**Figure 1:**
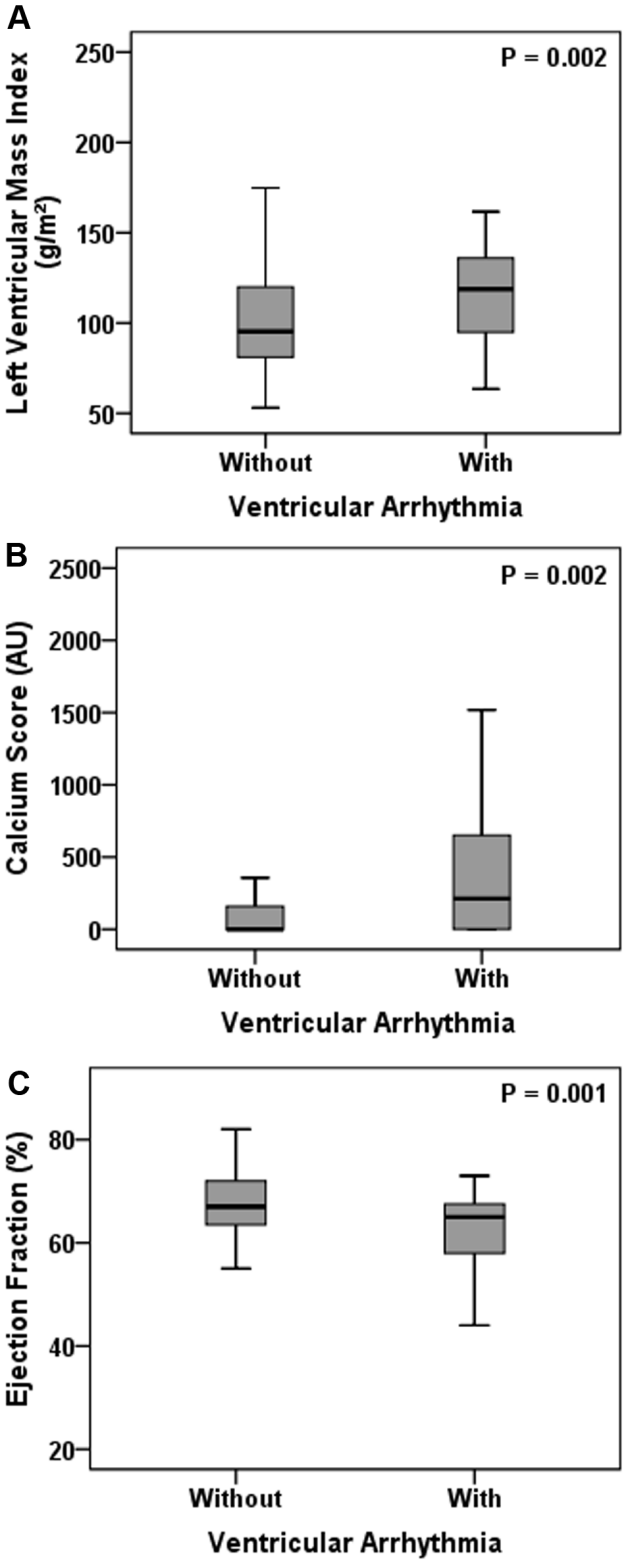
Cardiovascular parameters according to the presence of ventricular arrhythmia. Left Ventricular Mass Index (A), Calcium Score (B) and Ejection fraction (C) in patients with and without ventricular arrhythmia.

Ventricular arrhythmia was found in 39 patients (35%), from which 19 had also supraventricular arrhythmia. The median number of extra systoles in the population with ventricular arrhythmia was 51 (6–239) events/24 h. Table 2 depicts the comparison between patients with and without ventricular arrhythmia. Patients with ventricular arrhythmia were older, predominantly men, had higher eGFR and hemoglobin, and lower iPTH and triglycerides when compared to the patients without ventricular arrhythmia. Of note, eGFR correlated with hemoglobin (r = 0.422; p<0.01), iPTH (r = −0.51, p<0.01) but not with triglycerides (r = 0.16; p = 0.10). In addition, ventricular arrhythmia group had higher left ventricular mass index and coronary calcium score and lower ejection fraction (Figure 1).

**Table 2:** Comparison between patients with and without ventricular arrhythmia (VA).

	Without VA	With VA	p
**Number**	72	39	
**Male [n(%)]**	37 (51%)	30 (77%)	0.009
**Age (years)**	54±11	62±9.5	<0.001
**White [n(%)]**	42 (58%)	14 (36%)	0.07
**Follow up time (months)**	15.5 (8.2–55.5)	24 (11–55)	0.61
**Diabetes [n(%)]**	19 (26%)	8 (20%)	0.49
**Tobacco use [n(%)]**	33 (46%)	24 (61%)	0.11
**Body mass index (kg/m^2^)**	26.9±5.7	26.8±4.2	0.92
**Creatinine (mg/dL)**	2.4±0.9	1.97±0.67	0.007
**eGFR (ml/min/1,73 m^2^)**	32.4±15.9	39.5±15.8	0.03
**Proteinuria (g/24 h)**	0.37 (0–0.9)	0 (0–0.38)	0.02
**Hemoglobin (g/dL)**	12.4±1.8	13.4±1.61	0.005
**Potassium (mEq/L)**	4.8 (4.4–5.1)	4.7 (4.2–5.2)	0.30
**Magnesium (mEq/L)**	1.9 (1.72–2.1)	1.9 (1.7–2.1)	0.89
**Ionized calcium (mmol/L)**	1.27±0.05	1.28±0.05	0.34
**Phosphorus (mg/dL)**	3.85±0.74	3.6±0.68	0.18
**Alkaline phosphatase (mg/dl)**	78.5 (62–100.5)	87 (75–112)	0.07
**iPTH (pg/ml)**	132.5 (74.5–225.5)	94 (56–144)	0.02
**FGF 23 (pg/ml)**	45.4 (27.9–109)	63,1 (15.2–89.9)	0.68
**CRP (mg/dl)**	0.25 (0.09–0.69)	0.41 (0.15–0.85)	0.18
**IL6 (pg/ml)**	4.4 (2.2–8.5)	5.4 (3.0–8.0)	0.28
**Total cholesterol (mg/dL)**	185.6±37.7	181.3±38.03	0.57
**LDL cholesterol (mg/dL)**	101.3±30.1	100.4±24.4	0.87
**HDL cholesterol (mg/dL)**	51±13.1	52.8±17.7	0.53
**Triglycerides (mg/dL)**	139 (106–215.7)	110 (72–161)	0.01
**Median systolic pressure (mmHg)**	125.5 (117–138)	125 (115.7–134.2)	0.75
**Mean diastolic pressure (mmHg)**	79.2±10.7	77.7±11.3	0.50
**Absence of systolic decency [n(%)]**	21 (30%)	11 (29%)	0.56
**Left ventricular mass index (g/m^2^)**	95 (81–120)	119 (91–136)	0.002
**Ejection fraction (%)**	67 (63–72)	65 (58–68)	0.001
**Calcium score (AU)**	0 (0–168.7)	213 (1–671)	0.002

eGFR - estimated Glomerular Filtration Rate; iPTH - intact Parathyroid Hormone; FGF23 - Fibroblast Growth Factor 23; CRP - C-Reactive Protein; IL6 - Interleukin-6. Results in mean ± SD, median (interquartiles) or proportions.

When compared to patients without ventricular arrhythmia, those with ventricular arrhythmia exhibited higher frequency of systolic dysfunction (18% vs 6%; p = 0.037), ventricular hypertrophy (38% vs 21%; p = 0.047), and coronary artery calcification (69% vs 39%; p = 0.004).

In the stepwise logistic regression analysis, age, hemoglobin, and ejection fraction were the factors independently associated with the presence of ventricular arrhythmia in nondialyzed CKD patients (Table 3).

**Table 3:** Stepwise logistic regression analysis: Variables in the Equation.

Variables	Sig	Exp (B)	Lower 95%CI for Exp B	Upper 95%CI for Exp B
**Step 1**				
**Age**	0.001	1.076	1.030	1.124
**Step 2**				
**Age**	0.002	1.078	1.028	1.129
**Ejection fraction**	0.008	0.936	0.891	0.983
**Step 3**				
**Age**	0.004	1.076	1.024	1.130
**Ejection fraction**	0.008	0.936	0.891	0.983
**Hemoglobin**	0.021	1.391	1.052	1.839

Variables included in the model: age, gender, estimated glomerular filtration rate, proteinuria (g/24 h), hemoglobin, intact parathyroid hormone, triglycerides, left ventricular mass index, ejection fraction and calcium score.

## Discussion

According to the United States Renal Data System (USRDS) database, the single largest cause of death is attributed to arrhythmic disturbances. In fact, 26% of all-cause mortality among dialysis patients is due to cardiac arrest, unknown cause or arrhythmia [16]. The occurrence of ventricular arrhythmia and its associated risk factors had not been so far described in CKD patients in the initial stages of the disease. Herein we demonstrated that the prevalence of ventricular arrhythmia is elevated among CKD patients not yet requiring dialysis. In addition, we identified aging, hemoglobin levels and ejection fraction as the factors independently related to the presence of ventricular arrhythmia in these patients.

Patients with end-stage renal disease have several factors that could predispose to the development of ventricular arrhythmia. In the general population, the association of aging with episodes of fatal ventricular arrhythmia has been well recognized [17], [18]. Accordingly, in the present study, we confirmed the association of age with the occurrence of ventricular arrhythmia in patients with CKD. In fact, the aging process contributes to changes in the cardiovascular system such as increased arterial stiffness, increased systolic ventricular wall stress, and diastolic dysfunction [19]. Those structural cardiac alterations over time, along with the uremic cardiomyopathy, are potential contributors for the high prevalence of arrhythmias in CKD patients.

Numerous studies in the general population have pointed out men experience a higher rate of ventricular arrhythmia and sudden death when compared to women [20]–[22]. In patients with coronary artery disease and implantable cardioverter-defibrillators it has been demonstrated that women were less likely to experience ventricular tachycardia or ventricular fibrillation recurrences than men [20]. Accordingly, in the present study, 77% of the patients with ventricular arrhythmias were men. In fact, although the exact physiologic mechanism that triggers this phenomenon is not clear, it is likely that men have a greater propensity to ventricular arrhythmias than women [17]. It has been suggested that some differences in electrophysiologic properties related to sex hormones may, at least in part, explain the gender-specific propensity to ventricular arrhythmias [21], [23]. In addition, some studies advocate that gender differences in autonomic nervous system function, evaluated by variability in heart rate, could influence ventricular tachyarrhythmias [24], [25]. Actually, decreased heart rate variability frequently observed among men has been established as a significant risk factor for higher mortality in general population as well as in dialysis population [26], [27]. Corroborating with the above mentioned rationale, in the current study, a lower heart rate variability was observed more frequently among men when compared to women (14% vs 2%, p = 0.048, respectively).

In the present study, increased hemoglobin levels were independently associated with ventricular arrhythmia. Of note, few patients were on ESA therapy. Several previous studies, including CKD patients receiving ESA, on dialysis or not, have demonstrated that higher hemoglobin has no benefit [28], [29] or it is even associated with cardiovascular complications and greater risk of mortality [30], [31] in these patients. In a retrospective study with a cohort of 34,963 hemodialysis patients, each 1 g/dl increase in the residual standard deviation was associated with a 33% increase in the death rate [32]. Thus, a U-shaped relationship between hemoglobin levels and clinical outcomes has been suggested in this particular group of patients [33], [34]. More studies are necessary to explore the mechanistic explanation for these findings.

The traditional view of ventricular arrhythmia pathophysiology postulates a vulnerable diseased myocardium with a transient arrhythmic trigger [8], [9], [17]. Left ventricular hypertrophy and systolic dysfunction are highly prevalent in asymptomatic patients with end-stage renal disease, which sets a high background risk of arrhythmias in this population [7], [35]. The association between poor systolic function and ventricular arrhythmia or sudden cardiac death has been demonstrated in studies including both general [36], [37] and CKD [38], [39] population. Accordingly, a reduced ejection fraction was independently associated with the presence of ventricular arrhythmia in the present study.

Available literature suggests a relationship between left ventricular hypertrophy and cardiac arrhythmia in patients on hemodialysis [4], [5]. The myocardial fibrosis and hypertrophy provide additional substrate for an increased electric instability and may then contribute to an increased risk of ventricular arrhythmia and sudden cardiac death in uremic patients [37]. Paoletti *et al.* indicated that left ventricular hypertrophy, and particularly its progression, was the strongest predictive factor of lethal arrhythmias in uremic patients [40]. Of note, patients with ventricular arrhythmia in the present study had higher left ventricular mass index and a higher frequency of left ventricular hypertrophy.

It has been already established that coronary artery calcification is highly prevalent in dialysis patients as well as in nondialyzed CKD patients [41]–[44]. Not surprisingly, a high prevalence of coronary artery calcification was found in our study sample. We have previously demonstrated the straight association between vascular calcification and cardiovascular events in nondialyzed CKD patients [45], which is in line with the finding by other investigators [46], [47]. In the current study, coronary calcium score and the frequency of coronary artery calcification were both higher among patients with ventricular arrhythmia, when compared to those without this cardiac complication. The impact of the presence of ventricular arrhythmia on hard outcomes remains to be further investigated.

There is evidence in the literature that the protein-energy malnutrition might increases the risk of prolonged QT interval, ventricular arrhythmias and sudden death [48]. The only measure that could support this rationale herein is the lower triglycerides level found in the group of patients with ventricular arrhythmia. However, since only 4% of the patients in the current study were malnourished according to the subjective global assessment, this supposition is unlikely in this study. Another explanation for the lower triglycerides in the group with arrhythmias could be that these patients also have higher eGFR. However no correlation between these variables was observed in the present study. More studies are necessary to elucidate the physiopathology aspects involving such relationships.

Clinical studies in chronic dialysis patients have suggested a U-shaped relationship between PTH and sudden death, probably due to arrhythmia [49], [50]. In the present study, PTH levels were lower in patientes with ventricular arrhythmias. However, this group of patients had also better renal function, and unexpected findings were the higher eGFR and proteinuria in the group of patients with ventricular arrhythmia. According to the literature, both lower eGFR and the presence of proteinuria are associated with poorer cardiovascular outcomes in CKD patients [51]. Proteinuria has also been described as related to prolonged QT interval and other electrocardiographic abnormalities [52]. Thus, we cannot exclude the possibility of a survival bias due to the fact patients with worse renal function and ventricular arrhythmia may have passed away. Another possible explanation could be that eGFR does not accurately reflect the concentration of other different uremic solutes such as indoxyl sulfate, hippurate, and asymmetric dimethylarginine [53], [54], that are known to be linked to vascular damage and worse clinical outcomes [53].

This study has some limitations to be considered, such as the relatively small sample of prevalent CKD patients, what could introduce survival bias. Moreover, the cross-sectional design of the study does not allow us to evaluate the cause-effect relationship to derive conclusions.

In the present study, we concluded that ventricular arrhythmia was prevalent in nondialyzed CKD patients. Aging, increased hemoglobin levels and reduced ejection fraction were the factors independently associated with the presence of ventricular arrhythmia in these patients. To the best of our knowledge, this is the first study to evaluate the frequency of ventricular arrhythmia and its relationship with clinical, laboratorial and cardiovascular parameters in nondialyzed CKD patients. We believe that the present findings can contribute to improve the understanding in this field and draw attention to the need of an early diagnosis and treatment of ventricular arrhythmia during the nondialysis stages of the disease, in order to reduce its incidence and consequent sudden death rate in CKD population.
